# Psychiatric disorders and associated risk factors in a sample of adolescents in Gaborone, Botswana: a cross-sectional study

**DOI:** 10.1186/s12887-022-03435-7

**Published:** 2022-06-29

**Authors:** Anthony A. Olashore, Wendy Brooks, Hlanganiso Roy, Fatai Adewole Adebayo, Bonginkosi Chiliza

**Affiliations:** 1 Department of Psychiatry, Faculty of Medicine, University of Botswana, Gaborone, Botswana; 2 Department of Psychiatry, Nelson R Mandela School of Medicine, University of KwaZulu-Natal, Durban, South Africa; 3 Department of Statistics, University of Botswana, Gaborone, Botswana

**Keywords:** Psychiatric disorders, Adolescents, Botswana, Learners, Risk factors

## Abstract

**Background:**

Despite the high proportion of adolescents living with mental health issues in low- to middle-income countries (LMICs), especially in Botswana, there is a significant deficit of local research to guide an increase in prevention and treatment. We, therefore, aimed to assess the prevalence and associated risk factors of psychiatric disorders (PD) in a sample of secondary school students in Botswana.

**Methods:**

This cross-sectional study included 750 students from the 13 public secondary schools in Gaborone using a multi-stage sampling technique. The Mini-International Neuropsychiatric Interview for Children and Adolescents (MINI-KID) was used to screen for PDs.

**Results:**

The participant’s mean age was 15.26 and SD 1.57 years, with 53.6% being female. Approximately 34% had a PD, with depression being the commonest, of whom 35% were neither receiving treatment nor aware of the available services. Perinatal complications (AOR = 4.29; 95%CI: 1.04–17.70), a family history of mental illness (AOR = 2.19; 95%CI: 1.17–4.11) and substance-related problems (AOR = 1.80; 95% CI:1.22–2.65) predicted the likelihood of developing PD.

**Conclusions:**

Our findings revealed that adolescents in Botswana have many mental health issues which may affect their developmental phases. A multi-sectoral collaboration is needed for the timely detection of identified risk factors and initiation of the necessary prevention and treatment measures.

## Background

Adolescence is a critical stage of development that marks the period when most serious mental health issues begin [1]. This is when an individual undergoes numerous changes, including social, cognitive, and physical, from childhood to adulthood, which can significantly influence their mental health [2]. While this group accounts for 16% of the global population and up to 40% of some African countries [3–5], many African cultures still do not acknowledge that they may be under an immense amount of mental distress and need appropriate specialized mental health care [4].

Mental health disorders are a significant problem, relatively common, and treatable among adolescents. Despite the high proportion of adolescents in low- to middle-income countries (LMICs), most studies on the prevalence of mental disorders were done in high-income countries [4]. Therefore, there is a significant deficit of local research to guide the understanding of psychiatric disorders and their consequences on adolescent development in LMICs, particularly Botswana.

Approximately 10–20% of children and adolescents experience mental health problems worldwide [6]. A systematic review on the prevalence of mental disorders in sub-Saharan Africa (SSA) found that adolescents suffer predominantly from behavioral problems, depression, anxiety, posttraumatic stress disorder (PTSD), and suicidal ideations [4]. However, Botswana was not included in the review, possibly due to data on the community prevalence of specific mental health disorders being lacking.

While multiple factors have been shown to contribute to mental disorders, the most plausible scenarios would be the combination, sequence, and interaction of individual, familial, genetic, and environmental risk factors [7–9]. The demographic characteristics of individuals also have a significant bearing on the development of psychiatric disorders [3, 10]. For example, a study conducted in Brazil found that the prevalence of any psychiatric disorder was 30% (95%CI: 29.2–30.8) more common among girls than boys [11]. Externalizing disorders, such as attention deficit hyperactivity and conduct disorder, were higher in males, while internalizing disorders were more prevalent in females [3, 9, 10]. Findings on environmental risk factors revealed an inverse association between socioeconomic position and the probability of an individual developing a mental disorder. Low priority for mental health care and limited subject research to guide policies are also reported to be significant [3].

Mental disorders have a long-lasting impact on the child, their family, and society at large. These effects span from childhood to adult life [1, 10], and include poor school performance, delinquency, under-achievement in other aspects of their lives, unemployment, and the need for continuous disability support [1, 10]. Early recognition and prompt intervention have been shown to reduce mental health disease burden and improve the quality of life in children and adolescents [12]. However, the facilities and services to achieve this remain limited in Africa, including Botswana [3].

Botswana, a landlocked country in Southern Africa, is ranked third in the list of 47countries in SSA on the 2021 index of economic freedom, based on its property rights, judicial effectiveness, and government integrity [13]. Health care services are more than 60% government-funded, unlike other African countries, with mental health care services, especially for adolescents, being rudimentary. The only mental health facility with a relatively large number of specialists and a training program lacks adequate facilities for children with psychiatric problems. This may be due to the paucity of data to prove the existence of child psychiatric disorders as in this and other African countries [4]. While a previous study identified the presence of child and adolescents’ mental health problems in the only mental health referral institute in Botswana, there was no comprehensive community study to corroborate these findings. Thus, we contend that assessing the prevalence and associated risk factors of mental disorders in adolescents in Botswana could inform policy change favoring these services in Botswana.

## Methods

This was a cross-sectional study involving adolescents from age 12 to 19 years who were randomly selected from 13 of the 17 public secondary schools in Gaborone, Botswana, its inhabitants accounting for more than 10% of the country’s population. A sample size of 750 adolescents was arrived at using the Cochrane sample size estimation for an observational study and was selected from four senior secondary and nine junior secondary schools. ‘The Cochrane formula (N = Z2pq/d2) permits estimating an ideal sample size given a required level of precision, confidence level, and the projected proportion of the attribute present in the population. N’ is the minimum sample size required for the study. P = is the proportion in a previous study, d = Error margin (Precision) desired, set at 5% =0.05. Z = Standard normal deviate, set at 95% confidence level. (Z = 1.96). The estimated minimum sample was 373.3. We anticipated a non-response rate of 30%; hence we added 111.99. The estimated minimum sample size was (373 + 111.99) 485.29, approximating 500 participants, but we interview 750 students.

### Sampling technique

The sample frame was the list of 17 public secondary schools in the capital city of Botswana: 13 junior secondary schools and four senior secondary schools. We employed a multi-stage sampling technique, the first being a random selection of nine junior secondary schools out of thirteen and four senior secondary schools. This was done by compiling the list of the schools and entering them into a Statistical Package for Social Sciences (SPSS for Windows). The software was used to select the 13 schools out of 17. The second stage involved a random selection of classes from these schools. The Botswana junior schools have three grade levels, Forms 1–3, while the senior schools have Forms 4 and 5. In addition, all grade levels are further divided into several arms, as one level may have up to three or more classes, labeled ‘a’ ‘b’ ‘c’ or more. Therefore, the second stage involved a random selection of two to three classes, depending on the number of arms, while the third stage involved selecting eligible students from the class registers using SPSS. However, 300 participants were selected from the senior schools, which were fewer (75 students per school), while the remaining 450 were from the junior schools (50 students per school).

### Study procedure

The exercise was carried out with the help of five trained research assistants (RAs) who were all graduate students of Psychology. The RAs met the schoolteachers, who had been informed ahead of time, and discussed the study procedures with them. All the eligible students willing to participate were met during their lunch break or at a time specified as convenient for them. The issues regarding confidentiality and anonymity were also discussed with the consenting students. Letters regarding information about the study, consent forms, and parent sociodemographic questionnaire were sent to the parents of the eligible students, and only those who returned all the documents fully completed were enrolled in the study. Data collection took place from December 2019 to October 2020. The specified guidelines were followed for those whose interviews were conducted during the COVID-19 pandemic. All the participants with incomplete documents (i.e., completed sociodemographic and consent forms from parents) were replaced until the required sample size was achieved. Only those who returned these forms and assented were included in the study.

### Measures

Apart from the sociodemographic questionnaire designed by the authors based on the reviewed literature, three validated tools were used to assess the presence of mental disorders in the participants. First, the Diagnostic and Statistical Manual of Mental Disorders **(**DSM-5) criteria were used to assess the presence of substance a use disorder (SUD), followed by the General Health Questionnaire-12 (GHQ-12) to screen for psychological distress. Only those who scored a total of three or more on the GHQ-12 were regarded as having a high level of psychological distress and were further interviewed with the Mini-International Neuropsychiatric Interview for Children and Adolescents (MINI-KID). Finally, those positive for a psychological disorder were referred to the nearest clinic through class teachers or parents without breaching confidentiality.

**The sociodemographic** part of the questionnaire included questions about the respondents’ age, ethnicity, religious participation, parents’ marital status, level of education, and occupation. The parents’ occupation or employment status was classified based on the 2008 Botswana Standard Classification of Occupations [14], and it has nine groups. The nine groups were reorganized into three: 1) upper occupations (e.g., legislators, managers, professionals, large-scale traders). 2) lower occupations (e.g., plant operators, clerks, cleaners, small-scale traders, technicians) and 3) the unemployed.

**The Diagnostic and Statistical Manual of Mental Disorders (DSM–5)** [15] was used to assess substance use disorder (SUD). This version of DSM combines the DSM-IV categories of substance abuse and substance dependence into a single SUD. It comprises 11 criteria, of which two are required for a diagnosis. An individual was said to meet the criteria for SUD if he had used any psychoactive substance in the past 12 months and met at least two of the 11 DSM criteria as mentioned above.

**The General Health Questionnaire-12 (GHQ-12)** was used to evaluate psychological distress. This instrument has 12 items that assess the severity of psychological problems in the past few weeks. It is scored from 0 to 3 depending on the severity of the item being assessed, and the instrument generates a total score of 36. A score of 3 and above indicates psychological distress, based on the cut-off used in a previous study [16].

**The Mini-International Neuropsychiatric Interview for Children and Adolescents (MINI-KID)** [17] is a brief, structured, clinical diagnostic interview designed to assess the existence of 24 ICD-10 and DSM-IV mental disorders comprehensively and concisely. This interviewer-administered instrument is suitable for adolescents and has been used in various populations in African settings [18]. This tool has good internal consistency, as shown by the Cronbach’s alpha range, 0.82–0.89 [17]. It is organized into diagnostic sections or modules for various psychiatric disorders such as psychotic disorders, affective disorders, suicidality, anxiety disorder, and disruptive behavior disorders.

### Data analysis

The data were analyzed using the Statistical Package for Social Sciences (SPSS for Windows) Version 21. The descriptive statistics entailed: the mean to describe the continuous variables, such as age, the GHQ score, and the number of siblings, while percentages were used for the categorical variables, such as gender, religion, and ethnicity. The presence of any psychiatric disorder (APD), which is the outcome, was defined as meeting the criteria for at least one psychiatric disorder, as assessed by the MINI-KID. Chi-square tests were done to show the prevalence of psychiatric disorders by gender. Chi-square tests were also used to explore the relationships between APD and the categorical variables, such as gender, while the independent t-tests were performed to explore the relationship between the continuous variables such as age and APD. The significant variables on bivariate analysis were entered into a binary logistic regression model to further explore the relationships between the outcome and the dependent variables. A multi-collinearity test was performed to check for high inter-correlations among the predictors, and the Hosmer-Lemeshow goodness of fit test for logistic regression was also performed. The level of statistical significance for all tests was set at *p* < 0.05.

### Ethical considerations

The protocol was approved by the University of Botswana Research and Ethical Review Committee, Ministry of Health and Wellness, and Ministry of Education. Permission was also sought from the authorities of the selected school. Written informed consent was sought from all the parents of the selected students under the age of consent, while assent was obtained from the participants. The students who were 18 years and older were permitted to give consent, although their parents were informed.

## Results

Of the 750 secondary school students who participated in the survey, 742 (*n* = 98.9%) completed the questionnaire’s items on the variables of interest and were analyzed.

### Sociodemographic characteristics

The adolescent participants’ mean age was 15.26 (SD1.57) years, were mainly Christian (89.5%) from the Tswana ethnic group (80.6%), and more than half were females (53.6%) (Table 1). The majority (95.8%) claimed to have lost at least one parent to marital separation or death, of which 7.1% did so before they turned 5 years, and most could not tell when this happened.

**Table 1:** Demographic characteristics of the participants

Variable	Statistic	
	Mean (SD)	
**Age in years**	**15.26(1.57)**	
**Number of Siblings**	**2.0(1.0)**	
	**Frequency**	**%**
**Gender**	**738**^a^	**100**
Male	340	45.8
Female	398	53.6
**Religion**	**735**^a^	**100**
No religious affiliation	58	7.9
Christianity	658	89.5
Islam	4	0.5
Others^b^	15	2.0
**Ethnic group**	**713**^a^	**100**
Non-citizen	1	0.1
Tswana	575	80.6
Kalanga	100	14.0
Other ethnic groups^c^	37	5.2
**Loss of at least one parent to death or marital separation**	**736**^a^	**100**
No	18	2.4
Yes	718	96.8
**Age at parental separation or loss**	**86**	**100**
Less than age 5 year	53	7.1
More than age 5 years	33	4.4
**Care giver**	**712**	**100**
Both parents	124	16.7
Single parent and others	588	79.2
**Fathers’ occupation/employment status**	**596**^a^	**100**
Unemployed	125	21.0
Higher occupational level	237	39.8
Lower occupational level	234	39.3
**Mothers’ occupation/employment status**	**628**^a^	**100**
Unemployed	180	28.7
Higher occupational level	268	42.7
Lower occupational level	180	28.6
**Birth order**	**738**^a^	**100**
First born	176	23.7
others	562	75.7

^a^*N* = n not equal to 742 due to missing data, not applicable or don’t know

^b^Others = African traditional religion, and Hinduism and Buddhism

^c^Other ethnic groups such as Basarwa and Kgalagadi

### The pattern of psychiatric disorders and the clinical variables in the adolescents

**Fig. 1:**
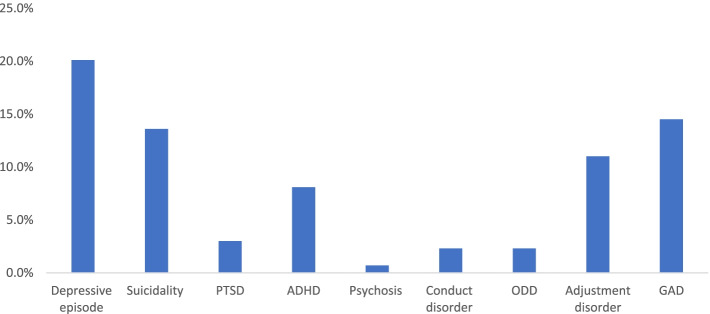
Prevalence of psychiatric disorders in a sample of adolescents in Gaborone, Botswana

All 742 adolescents were screened for psychiatric disorders, and 256 (34.5%) met the diagnostic criteria of at least one psychiatric disorder (APD). Of the 256, parents of 85 adolescents were aware that mental illness is treatable, while 91 were aware of the available treatment services but unaware of these conditions in their children. Depression was the most common, accounting for 20.1% of the psychiatric disorders, followed by generalized anxiety disorder and suicidality (Fig. 1). Substance use disorder (31.6%) and its associated factors have been reported in another manuscript. Depression (χ^2^ = 11.3, *p =* 0.01), suicidality (χ^2^ = 10.6, *p* = 0.01) and adjustment disorder (χ^2^ = 7.21, *p* = 0.007) were significantly more common in females, while the externalising disorders such as ADHD (χ^2^ = 6.39, *p =* 0.011), CD (χ^2^ = 4.21, *P* = 0.040), and ODD (χ^2^ = 4.21, *P* = 0.040) were more common in males (Table 2).

**Table 2:** Frequency of the diagnosis by gender

Frequency of diagnosis	Gender			
	Male N (%)	Female N (%)	Chi-square	***P*** value
Depressive episode	50(6.8)	98(13.3)	11.3	**0.01**
Suicidality	31(4.2)	69(9.3)	10.6	**0.01**
PTSD	6(0.8)	16(2.2)	3.25	0.073
Substance use disorder	121(16.4)	112(15.2)	4.71	**0.030**
ADHD	37(5.0)	23(3.1)	6.39	**0.011**
Conduct disorder	12(1.6)	5(0.7)	4.21	**0.040**
Psychosis	3(0.4)	2(0.3)	0.39	0.666*
Adjustment disorder	26(3.5)	55(7.5)	7.21	**0.007**
ODD	12(31.2)	5(68.8)	4.21	**0.040**
GAD	51(6.9)	56(7.6)	0.12	0.721

*Fisher’s exact test, *ODD* Oppositional defiant disorder; *ADHD* attention deficit hyperactivity, Psychosis = schizophrenia and other psychotic disorders, *PTSD* Post traumatic stress disorder; *GAD* Generalized anxiety disorder, N^*^ = 238. Significant relationships in Bold Print

Perinatal complications were reported concerning 23 (3.1%) of the participants; 60 (8,1%) were reported to suffer from early childhood medical disorders, such as epilepsy, while 84 (11.3%) confirmed positive family history of mental illness. According to the parents’ reports, 20 (2.7%) of the adolescents have been previously diagnosed with a mental illness, with nine not knowing what it was. Out of the 20 participants whose parents confirmed a diagnosis of mental illness, only ten were currently receiving treatments, the remainder neither being aware that the illness can be treated nor that services for mental health care exist in Botswana.

### Bivariate analysis of the associated risk factors of psychiatric disorders

The bivariate analysis indicated that the history of early childhood medical illness, perinatal complication, mental illness in the family, and SUD were significantly associated with APD (Table 3). In addition, the type of caregiver, such as single-parent or other relatives or friends (χ^2^ = 4.18 *P* = 0.041), fathers’ employment status (χ^2^ = 6.55, *p* = 0.038), and age (t = − 2.39 *p* = 0.017) were significantly associated with APD.

**Table 3:** Associated factors of any psychiatric disorder in the participants

Variables	Psychiatric disorder				Statistic		
	Absent		Present		***df***	***χ***^**2**^	*p*
	Frequency	%	Frequency	%			
**Gender**							
Male	225	66.2	115	33.8	1	0.21	0.648
Female	257	64.6	141	35.4			
**Ethnic group**							
Non-citizen	-	–	1	100.0	3	5.13	0.16
Tswana	367	63.8	208	36.2			
Kalanga	73	73.0	27	27.0			
Other ethnic groups	25	67.6	12	32.4			
**Religion**							
No religious affiliation	46	79.3	12	20.7	3	6.36	0.095
Christianity	424	64.4	234	35.6			
Islam	3	75.0	1	25.0			
Others	8	53.3	7	46.7			
**Birth order**							
First Born	112	63.6	64	36.4	1	0.34	0.56
Others	371	66.0	191	34.0			
**Loss of at least one parent to death or marital separation**							
Absent	10	55.6	8	44.4	1	0.79	0.374
Present	470	65.6	246	34.4			
**Age at parental separation or loss**							
5 years and above	20	60.6	13	39.4	1	0.49	0.480
Less than 5 years	28	52.8	25	47.2			
**Care giver**							
Both parents	91	73.4	33	26.6	1	4.18	**0.041**
Others^a^	375	63.8	213	36.2			
**Fathers’ occupation or employment status**							
Unemployed	73	58.4	52	41.6	2	6.55	**0.038**
Higher occupational level	152	64.1	85	35.9			
Lower occupational level	167	71.4	67	28.6			
**Mothers’ occupation or employment status**							
Unemployed	111	61.7	69	38.3	2	1.14	0.57
Higher occupational level	176	65.7	92	34.3			
Lower occupational level	120	66.7	60	33.3			
**Early childhood illness**							
Absent	459	67.3	223	24.2	1	12.2	**< 0.01**
Present	27	45.0	33	55.0			
**Perinatal complication**							
Absent	480	66.9	238	33.1	1	20.1	**< 0.01**
Present	5	21.7	18	78.3			
**Family history of mental illness**							
Absent	450	68.4	208	31.6	1	21.4	**< 0.01**
Present	36	42.9	48	57.1			
**Substance related problems**							
Absent	358	70.5	150	29.5	1	17.6	**< 0.01**
Present	128	54.7	106	45.3			

Significant *p* values in bold, ^a^Others: Single parent and others

### Binary regression analysis of the associated risk factors of psychiatric disorders

Table 4 indicates that all the significant variables on bivariate analysis were entered into a regression model. Participants with a history of Perinatal complications were four times more likely to develop APD (Odd ratio (OR) = 4.29; 95% CI: 1.04–17.70), while those with a family history of mental illness were twice as likely to develop APD (OR = 2.19; 95%CI: 1.17–4.11). Substance use disorder also predicted the likelihood of developing APD in the participants (OR = 1.80; 95% CI:1.22–2.65).

**Table 4:** Logistic regression analysis of significant variables

Characteristics	Wald	Sig	AOR	95% CI. for Exp (B)	
				Lower	Upper
**Single parent and others**	0.89	0.343	0.68	0.31	1.50
**Higher occupational level**	2.28	0.131	0.68	0.41	1.12
**Older age**	3.65	0.056	1.13	0.99	1.29
**Early childhood illness (present)**	1.25	0.264	1.52	0.73	3.19
**History of Perinatal complication**	4.07	**0.044**	4.29	1.04	17.70
**History of Substance related problems**	8.86	**0.003**	1.80	1.22	2.65
**Family history of mental illness**	5.99	**0.014**	2.19	1.17	4.11

Significant *p* values in bold

## Discussion

In this study, 34.5% of the secondary school students met the diagnostic criteria of at least one disorder. This supports the earlier assertion that psychiatric disorders are also present among students in Botswana [3] and indicates that they are highly prevalent in the community, just like in upper-income countries [19] and LMICs [4]. It further confirms the earlier claims that there is a significant unmet need among the adolescents in Botswana, given that approximately 35% of those diagnosed with psychiatric disorders were neither receiving treatment nor aware of the available psychiatric care services. In addition, the fact that most of the schools visited for data collection had counselors is an indication that the available services were grossly underutilized. This suggests the need for community awareness about mental disorders, as most can be identified by immediate caregivers rather than the school care services, if they are well educated and empowered.

In accord with the literature [10, 20–22], our study revealed a higher prevalence of internalizing disorders than other psychiatric disorders. However, it is noteworthy that studies conducted in special populations, such as child welfare services and hospitals, reported more externalizing symptoms than internalizing disorders [3, 19]. This may be explained by the fact that those with externalizing symptoms are more likely to be absent from schools, present at the hospital, or more likely to require social services. Therefore, there is a need to actively screen for internalizing disorders in schools and communities. This exercise has become necessary as internalizing disorders, such as depression and anxiety, are associated with high mortality in the form of suicide or deliberate self-harm [23]. The pattern of psychiatric disorder in the present study regarding gender follows the existing trends [3, 20]. We observed a preponderance of externalizing disorders, such as ADHD, CD, and ODD in male participants, and internalizing disorders in the females, suggesting what to look for in various settings.

The findings of our study are consistent with other studies that have identified the harmful effects of Adverse Childhood Experiences (ACEs) on the adolescent’s developmental pathway [24]. In addition, the bivariate analysis indicated a relationship between mental disorders and some ACEs, such as early childhood medical conditions, perinatal complications, and being raised by a single parent or other relatives or friends.

Generally, a stable family with both parents can moderate a smooth transition from childhood through adolescence to adulthood, as both parents can contain the challenges and support the child. The absence of a parent can create a void or gap in the child’s developmental pathway [25], and much confusion typically follows as the child seeks to make sense of the loss or absence while also struggling with self-assertion and identity formation. When this is not well managed or contained, the adolescent is at risk of developing mental health complications, such as depression, anxiety, or falling into substance use. Nevertheless, this dysfunctional upbringing and other factors, such as the possible effects of childhood illness on brain development and poor socioeconomic factors, as shown by the father’s employment status, failed to predict the same after logistic regression. Conversely, other risk factors, such as a family history of mental illness, perinatal complication, and substance-related problems, continued to predict mental disorder after a binary logistic regression, thus suggesting an interplay between nature and nurture.

Studies have established the association between a family history of mental disorders and an increased risk of developing various psychiatric disorders [26, 27]. One example is a study that related reduced cognitive ability with a family history of mental disorders [26], with affected participants being twice as likely to develop APD. While most of the parents did not indicate the type of disorder in the family member, a few mentioned disorders that were unrelated to the diagnosis made in their children. Regardless, this finding suggests the benefit of routine screening of family members of individuals living with psychiatric disorders. This practice is becoming very popular as it enables the necessary steps to be taken toward early detection and prevention of mental disorders, especially severe ones [28]. As nurture plays a significant role as a precipitator in those highly vulnerable by nature (i.e., high genetic load), advice or education on healthy living may play a huge role in prevention and mental health promotion.

Participants whose parents reported a perinatal complication were four times more likely to develop APD in the present study, with such complications and mortality rates having remained unacceptably high in Africa [29]. Most of the countries in SSA have failed to meet the MDGs on maternal and child survival [29]. With more than 60% of health care services being government-funded, Botswana is not excluded from these problems, as up to 50% of neonatal deaths were related to preterm birth complications and intrapartum-related events in 2017 [30]. A systematic literature review revealed that mothers who experience gestational diseases, including drugs and infection, had more than a three-fold increased risk of having children with psychiatric disorders [31]. In the same vein, those who experienced perinatal complications had a two-fold increased risk of giving birth to children with mental illness [31]. Our findings support the neurodevelopmental hypothesis of mental disorder in that events occurring within the intrauterine or perinatal environment at critical times of brain development underlie the emergence of psychiatric disorders observed in adulthood [31]. Hence, addressing the identified causes of perinatal complications in Botswana, such as home delivery, poor antenatal care (ANC) attendance, and unorthodox alternative care [32], might contribute to decreasing mental disorders.

As adolescents weave through the transition to adulthood, they face many competing factors. These include the pursuit of self-identity and independence, which are premised on peers or other models’ perceived “good standing” [33]. As previously alluded to, these often require physical resources, such as financial stability and a stable/supportive family structure. When these fail, the peer pressure could overwhelm and push them into substance use and, ultimately, mental disorders. Approximately 31% of the participants admitted to having substance-related issues, and a little less than half of these met the criteria for one psychiatric disorder. Additionally, substance-related problems predicted the likelihood of developing APD in the participants.

Though poorly defined, the relationship between substance use and psychiatric disorder is bidirectional. Studies have found that approximately 50% of those who experience a mental illness during their lives will also experience a substance use disorder or have experimented with psychoactive substances and vice versa [34, 35]. The nature versus nurture theory suggests that substance use can precipitate APD in individuals with a silent or inactive gene for mental disorders [36]. This theory is supported by the role of epigenetic modification, known as DNA methylation, in the development and course of psychiatric disorders [36]. For example, Arseneault and colleagues have calculated that removing cannabis from the community would prevent approximately 8% of psychosis [37]. Therefore, it is possible that campaigns and other initiatives targeted toward drug eradication can significantly reduce the rate of new mental disorders in our community.

## Limitations

The association observed between the identified risk factors and psychiatric disorders appears interactive rather than unidirectional. This being the case, youth-targeted interventions should also be multi-layered, as not only one causality is at play but varied factors. The specific configurations of these factors were observed, and their interplay, the extent to which they interact and eventually lead to APD for some and sparing others, is not testable within the available data of this research study. However, noting the trajectories by which exposure to these factors creates resilience or vulnerability on others requires further exploration.

This study was done in one urban locality, which may be a limitation. An expansion to more localities could yield results to represent the cross-spectrum of the adolescents across the country, such as urban, semi-urban, and rural areas, and inclusive, diverse populations, such as those not in schools. In this way, the socio-cultural and economic dimensions of the challenges could be better illuminated. In addition, the anxiety relating to the COVID-19 pandemic may also have affected our findings, as some of the samples were collected during the early period. Nonetheless, the use of a large number of adolescents from the largest heterogeneous city in Botswana, a rigorous methodology, and standardized tools were the strengths of this study.

Potential sources of bias include:

- Recall bias, as most of the questionnaires used in the study require the participants to recall symptoms or events that occurred in the past. This is especially possible among those with depressive symptoms.
- Biased symptom reporting, which is possible in those with the diagnosis of behavioral disorders.
- Selection bias, as the sample selection was restricted to the capital city of Botswana; consequently, the results may not be generalizable to other districts. Hence, we recommend a cautious interpretation of our findings.

## Conclusion

The results provide evidence that adolescents in Botswana have many challenges, which may be affecting their developmental phases, from as early as perinatal to adolescence, and could spill over into adulthood. Furthermore, it is worrisome that many parents do not know that mental health services exist in their communities. We thus conclude and note the observation made by Schulte-Körne [6] that Physicians should collaborate with school social workers and psychologists to help teachers recognize and contend with mental health problems among the adolescents they teach. In addition, it will enable the timely detection of stress factors at school and the initiation of the necessary measures and aids. Activities and policies that promote the mental health of these young people, such as regular media chats on mental health, aggressive community outreaches, and campaigns against illicit drug use and poor ANC, should also be encouraged.

## Data Availability

The datasets used and analyzed during the current study are available from the corresponding author on reasonable request.

## Abbreviations

ADHD: Attention deficit hyperactivity disorder
ACEs: Adverse Childhood Experiences
ANC: Antenatal care
APD: Any psychiatric disorder
DSM-5: Diagnostic and Statistical Manual of Mental Disorders, fifth edition (DSM-5)
IRB: Independent Review Board
LMICs: Low to middle-income countries
ODD: Oppositional defiant disorder
PD: Psychiatric disorders
PTSD: Posttraumatic stress disorder
